# Integrating consumer perspectives into a large-scale health literacy audit of health information materials: learnings and next steps

**DOI:** 10.1186/s12913-023-09434-3

**Published:** 2023-04-29

**Authors:** Julie Ayre, Carissa Bonner, Jemma Gonzalez, Teresa Vaccaro, Michael Cousins, Kirsten McCaffery, Danielle M. Muscat

**Affiliations:** 1 Sydney Health Literacy Lab, Sydney School of Public Health, Faculty of Medicine and Health, The University of Sydney, Rm 128C Edward Ford Building, Sydney, NSW Australia; 2 Menzies Centre for Health Policy and Economics, Sydney School of Public Health, Faculty of Medicine and Health, The University of Sydney, Sydney, Australia; 3 NPS MedicineWise, Sydney, Australia

**Keywords:** Health literacy, Organisational health literacy, Health information, Plain language, Patient education, Health education, Consumer health information, Consumer engagement, Consumer involvement

## Abstract

**Background:**

Health information is less effective when it does not meet the health literacy needs of its consumers. For health organisations, assessing the appropriateness of their existing health information resources is a key step to addressing this issue. This study describes novel methods for a consumer-centred large-scale health literacy audit of existing resources and reflects on opportunities to further refine the method.

**Methods:**

This audit focused on resources developed by NPS MedicineWise, an Australian not-for-profit that promotes safe and informed use of medicines. The audit comprised 4 stages, with consumers engaged at each stage: 1) Select a sample of resources for assessment; 2) Assess the sample using subjective (Patient Education Materials Assessment Tool) and objective (Sydney Health Literacy Lab Health Literacy Editor) assessment tools; 3) Review audit findings through workshops and identify priority areas for future work; 4) Reflect and gather feedback on the audit process via interviews.

**Results:**

Of 147 resources, consumers selected 49 for detailed assessment that covered a range of health topics, health literacy skills, and formats, and which had varied web usage. Overall, 42 resources (85.7%) were assessed as easy to understand, but only 26 (53.1%) as easy to act on. A typical text was written at a grade 12 reading level and used the passive voice 6 times. About one in five words in a typical text were considered complex (19%). Workshops identified three key areas for action: make resources easier to understand and act on; consider the readers’ context, needs, and skills; and improve inclusiveness and representation. Interviews with workshop attendees highlighted that audit methods could be further improved by setting clear expectations about the project rationale, objectives, and consumer roles; providing consumers with a simpler subjective health literacy assessment tool, and addressing issues related to diverse representation.

**Conclusions:**

This audit yielded valuable consumer-centred priorities for improving organisational health literacy with regards to updating a large existing database of health information resources. We also identified important opportunities to further refine the process. Study findings provide valuable practical insights that can inform organisational health actions for the upcoming Australian National Health Literacy Strategy.

**Supplementary Information:**

The online version contains supplementary material available at 10.1186/s12913-023-09434-3.

In 2021 the World Health Organization (WHO) revised their definition of health literacy to emphasise the importance of the health literacy environment, in addition to an individual’s capacity to access, understand, appraise, and use health information and services [1]. This framing shifts the onus of addressing health literacy from individuals to the health systems themselves, and advocates for health systems that are responsive and accessible to patients.

Health literacy at a systems level is called ‘organisational health literacy.’ This encompasses initiatives that make it easier for people to navigate, understand and use information and services provided by an organisation or health system. Organisations may also seek to build individual and community health literacy skills, though this is less common [2]. Despite an increasing interest in organisational health literacy and a wide array of relevant theories, frameworks, and guidelines, there has been limited research investigating how to operationalise and implement strategies to address organisational health literacy [3].

A clear example of the gap between organisational health literacy theory and practice is the limited availability of plain language health information. Providing health information that people can easily understand and act on is central to almost all health literacy policies, frameworks and guidelines [4]. Calls to improve the quality of health information are not new. Over a decade ago, China released the 2008 ‘National Plan of Health Literacy Promotion Initiatives’ [5] and the US launched the 2010 National Action Plan to Improve Health Literacy, the first goal of which was to develop simpler health information [6]. Similar national policy documents appeared across Australia, New Zealand, Austria, and Scotland in subsequent years [4]. And yet, health information continues to be communicated in a manner that is too complex for many in our community. For example, in Australia online health information typically averages 2 to 5 school reading grades above the recommended level of grade 8 [7–9].

Many health literacy guidelines recommend partnering with consumers when developing health information [9–12]. The benefits of consumer engagement are documented empirically by systematic reviews of the broader healthcare literature, showing improved outcomes for health care, service delivery, policy, and health education [13, 14]. However, within the domain of health literacy, this research is relatively sparse. Mastroianni and colleagues [15] provide a promising example. They evaluated a new health information approval process requiring that all new health information follow plain language guidelines (e.g. documents written at a grade 8 reading level or lower) and incorporate feedback from at least 5 consumers. This process was implemented within a regional health service in New South Wales, Australia [15, 16]. Pre-post testing of 50 documents showed that these requirements significantly improved independent ratings of how easily the health information could be understood and acted upon.

For many organisations it is unclear how to best implement plain language guidelines and increase consumer engagement when there is a large *existing* library of health information and limited resources with which to revise the information. In such cases, an ‘audit’ of a sample of resources can give some insight into potential systemic issues across resources. For example, Alpert, Desens [17] used the CDC Clear Communication Index to assess the health literacy of the 37 most frequently visited webpages from a popular US patient portal. By using this validated health literacy assessment tool, the authors were able to clearly identify that patient portal resources could be improved by using simpler language, more specific examples to illustrate concepts, and clearer numerical explanations. However, the audit lacked any consumer perspective, limiting the opportunity for more meaningful interpretation of audit findings. Incorporating the consumer perspective aligns with the World Health Organization’s recent recommendation to involves service users in organisational audits and embed co-design into health literacy responsive health systems [12].

To date, no research about health literacy audits for large databases of existing health information has integrated a consumer perspective. This study aimed to describe novel methods for a consumer-centred large-scale health literacy audit of existing resources developed by an Australian not-for-profit organisation, NPS MedicineWise. A secondary aim was to explore the experiences of staff and consumers who engaged in the audit, to highlight opportunities for further improvement to the audit method.

## Methods

### Study setting and design

NPS MedicineWise is a national consumer-centred Australian not-for-profit organisation that promotes the safe and wise use of medicines and other health technologies. The NPS MedicineWise website contains online versions of official consumer medicine information (CMI) as well as online resources to support safe and appropriate use of medicines and health technologies, education about various health conditions and tools to support health behaviours and informed decision-making (e.g. action plans and patient decision aids).

This study focuses on an audit of existing consumer resources, education and tools (collectively described as resources for this article) developed by NPS MedicineWise through the federally funded *Quality Use of Diagnostics, Therapeutics and Pathology Program* (Australian Government Department of Health and Aged Care). The audit did not include CMIs as these are developed externally by medicine sponsors and manufacturers in accordance with Therapeutic Goods Administration (TGA) regulations for registered medicines. Ethical approval was obtained from the University of Sydney Human Ethics Committee (Project number 2022/153). This committee ensures that research is conducted within the guidelines set out in the Australian National Statement on Ethical Conduct in Human Research (2007) – Updated 2018. After reading the participant information statement, interested participants then indicated informed consent via completion of the survey. Consumers were involved throughout project planning and implementation.

**Fig. 1:**
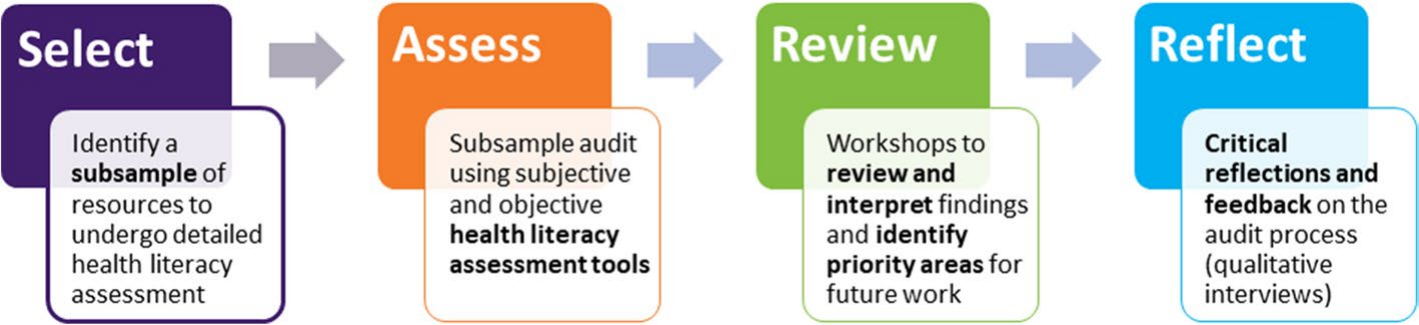
The four stages of the audit process

The audit process comprised 4 stages (Fig. 1), each described in further detail below.

#### Stage 1: Selecting a sample of resources

The NPS MedicineWise audit identified 147 individual consumer resources including web-based articles, downloadable factsheets and shared-decision making tools, and videos. Five consumers attended a workshop (Workshop A) to identify a sample of these resources (*n* = 49) for further auditing. To facilitate this task, the consumers were presented with data summarising the resources, including general descriptive data (e.g. health topic; resource type such as standard written content, audio-visual or fact sheet) and user data (e.g. unique visits, time spent on page).

In addition, consumers were presented with data showing specific health literacy skills addressed in each resource. NPS MedicineWise previously collaborated with the Consumers Health Forum of Australia (Australia’s leading advocacy group for consumer health care issues) to develop Health Literacy Quality Use of Medicines Indicators [18]. The indicators encompass 5 domains of health literacy skills relevant to quality use of medicines: individual health literacy, understanding quality use of medicines, engaging with health professionals, reading medicine information, and accessing further information. Development of the indicators (herein referred to as ‘health literacy skills’) was informed by the literature, consumer-led online discussion forums with 185 consumers, and survey responses from 1,503 consumers.

In selecting the 49 resources for further auditing, consumers and staff were asked to consider the need for a variety of resources, including those with low and high webpage visits, and low and high coverage of health literacy skills. If consumers identified other reasons for prioritising a resource for further auditing, these were also integrated into the selection process.

#### Stage 2: Health literacy assessment of the sample

Study authors prioritised health literacy assessment tools if they were widely used within health literacy research and practice, provided numeric output, and would be feasible to implement. A combination of objective and subjective assessment tools was sought, with subjective assessments carried out by four of the consumers who selected the resources in stage 1. The tools are underpinned by a universal precautions approach to health literacy, which argues that all patients and caregivers benefit from health information that is easier to understand [19].

##### Patient Education Materials Assessment Tool (PEMAT)

The PEMAT [20] was selected because it is a validated and widely-used tool to assess the health literacy demands of a given resource. The tool consists of 26 items and provides assessment of two domains. The first domain, understandability, refers to how easily readers of varying health literacy levels can process and explain a text’s key messages. It comprises five topics: content; word choice and style; medical terms; numbers; organisation; layout and design. The second domain, actionability, relates to how easily a reader can identify what they can do. Assessments for each domain are presented as a percentage, with scores ≥ 70% considered adequate.

Consumers received PEMAT training including practising on three ‘test’ resources, with the opportunity to reflect on the task as a group and ask further questions. Two consumers then independently rated each resource using the PEMAT. Once all resources were assessed, any discrepancies were resolved through discussion between the pair of consumers.

##### Sydney Health Literacy Lab (SHeLL) Health Literacy Editor

The SHeLL Health Literacy Editor (the Editor) is an automated online tool that provides real-time feedback on the complexity of health information [21]. It was selected because it could provide objective assessment beyond grade reading score. The two additional assessments used in this study were complex language and passive voice.

Grade reading scores are widely used in health literacy research to estimate text complexity in relation to school grade reading levels. The Editor uses a readability formula called the Simple Measure of Gobbledygook (SMOG) [22]. This formula is a more reliable, robust, and conservative estimate of grade reading score compared to other readability formulas [23–25]. In Australia, health literacy guidelines recommend that information is written at a grade 8 reading level or lower [9].

The complex language score reports the proportion (as a percentage) of words in the text being assessed that are flagged by the program as ‘complex.’ This includes acronyms, any words for which a simpler alternative has been identified, based on public health and medical thesauruses, and any words that are flagged as ‘uncommon’ in English, according to a database of more than 270 million words. Although there are no specific targets for complex language, lower scores are considered easier to understand as they contain fewer complex words. For this project, a target of < 15% complex language was used.

The passive voice score indicates the number of passive voice constructions in the resource (e.g. passive voice: *the medicine was given to the patient;* active voice: *the doctor gave the medicine to the patient*). In line with the PEMAT, resources should use no more than 1 instance of passive voice.

NPS MedicineWise staff assessed grade reading score, complex language, and passive voice for the 49 online resources using the Editor. Data were collated in preparation for Stage 3.

#### Stage 3: Workshops to review and interpret audit results, and identify priority areas

NPS MedicineWise staff and consumers were invited to attend two online workshops. The aim of these workshops was to present the results of Stage 1 and 2 of the audit and facilitate discussions to establish recommendations for revising, creating, and removing online content. The five consumers involved in Workshop A (Stage 1) were invited to take part in Stage 3, in addition to other NPS MedicineWise consumers. Workshop content and activities were designed in collaboration with and facilitated by author MC, a consumer representative with a long-standing relationship with NPS MedicineWise and chair of the NPS MedicineWise consumer advisory group. Materials were distributed prior to the workshops to provide background reading and audio-visual content explaining the project and audit findings. The first of these workshops (Workshop B) focused on presenting the background to the study and audit findings, with the goal of interpreting the audit findings collectively, as a group. The second (Workshop C) focused on identifying potential areas for improvement with small groups looking at specific resources.

#### Stage 4: Critical reflections on the audit process

Attendees from the latter workshops (B and C) were invited to take part in semi-structured interviews. Interview questions asked for feedback on the health literacy audit methods and suggestions for further improvement. After obtaining consent, author JA interviewed participants via Zoom individually or in small focus groups. Participants could comment on any part of the health literacy audit. Audio data were transcribed and feedback collated. Participants were interviewed between 25^th^ May 2022, and 9^th^ June 2022.

## Results

### Stage 1: Selecting a sample of resources

Data about each of the 147 resources were presented to consumers (Appendix 1). Of these, 47 (32.0%) provided general information about quality use of medicines and 29 (19.7%) were about pain and pain medicines. The remaining categories covered topics such as heart health, COVID-19, dementia, and bone health. For resources with available user data, the median page visits per resource was 1,662 in 2019–2020 (interquartile range (IQR) = 3,113), and 1,604 in 2020–2021 (IQR = 2,845). Median time spent on a resource was 2 min 41 s in 2019–2020 (IQR = 1 min 54 s), and 2 min 33 s in 2020–2021 (IQR = 2 min 6 s).

A summary of how frequently health literacy skills appeared is presented in Table 1. Across all resources (*N* = 147), the health literacy skills that featured most often were those that encouraged users to ask health professionals questions about their medicines (*n* = 100, 68.0%), think about the benefits and risks of a medicine (*n* = 94, 63.9%), seek advice from a health professional before starting medicine (*n* = 86, 58.5%), and read about medicine side effects on medicine labels (*n* = 85, 57.8%).

**Table 1:** Health literacy quality use of medicines indicators (health literacy skills) included in all resources (*N* = 147) and selected sample of resources (*n* = 49)

Resource encourages reader to…	All resources		Selected resources	
	**n**	**%**	**n**	**%**
**Individual health literacy**				
Ask questions to seek clarification	77	52.4	22	44.9
Seek further information	72	49.0	30	61.2
Discuss issues relating to complementary medicines	46	31.3	19	38.8
Discuss issues relating to cost of medicines	10	6.8	5	10.2
**Understanding quality use of medicines**				
Think about benefits and risks of a medicine	94	63.9	34	69.4
Think about medicine interactions	43	29.3	20	40.8
Think about medicine addiction	10	6.8	5	10.2
Store medicine correctly	10	6.8	5	10.2
Dispose of medicine safely	7	4.8	4	8.2
Avoid taking another person’s prescription medicine	7	4.8	4	8.2
Check a medicine’s expiry date	2	1.4	2	4.1
**Engaging with health professionals**				
Ask the health professional questions	100	68.0	31	63.3
Seek professional support before starting medicine	86	58.5	30	61.2
Engage in shared decision making	74	50.3	28	57.1
Prepare for health consultations	45	30.6	16	32.7
Use the same health professional (for people with ongoing health issues)	5	3.4	2	4.1
**Reading medicine information**				
Learn about a medicine’s side effects	85	57.8	30	61.2
Learn about the medicine dose	61	41.5	25	51.0
Check the medicine’s active ingredient(s)	50	34.0	20	40.8
Read the medicine label	44	29.9	15	30.6
Learn what the medicine is used for	40	27.2	14	28.6
Read pharmacist instructions on the medicine	34	23.1	13	26.5
Learn about the medicines warning/allergies	24	16.3	9	18.4
**Accessing further information**				
Seek information from a telephone information service	45	30.6	18	36.7
Seek information in the Consumer Medicines Information (CMI) Leaflet	35	23.8	11	22.4

The health literacy skills that featured least often (in less than 10% of resources) were those relating to medicine expiry dates, disposal, storage, cost, and addictiveness; taking others’ prescription medicines; and advice to have a consistent health professional. On average each resource covered 17 of the 25 health literacy skills (SD = 3.9).

During Workshop A consumers identified 49 resources for more detailed audit. An additional resource had already been identified by NPS MedicineWise staff and used as an example to support discussions during this workshop.

The detailed audit included resources from each key health topic available on the NPS MedicineWise website, and across all formats (e.g. standard written content and audio-visual formats). Table 1 shows that each health literacy skill featured at least once in the selected resources. Throughout the selection process, consumers and staff used the summary data in conjunction with broader criteria e.g. making sure that resources related to different lifespan stages (i.e. childhood), specific topics of interest (e.g. managing migraine), and COVID-19.

### Stage 2: Health literacy assessment of the sample

Consumer ratings of PEMAT items are presented in Table 2. Overall, 42 of the resources (85.7%) had adequate understandability. Within this domain, all resources were rated as presenting information in a logical sequence (100%) and having informative headers (100%). Almost all resources scored high on items related to word choice and style (range 94%-98%) and for most resources the visual aids (when present) were clear and uncluttered (97%) and reinforced the written content (92%). Few resources provided a summary (27.9%), and only one third used visual aids whenever possible (32.5%).

**Table 2:** Endorsement of PEMAT items, selected resources (*n* = 49)

PEMAT item	n endorsed	n eligible (denominator)^a^	%
**Understandability**^b^	**42**	**49**	**85.7**
**Content**			
Makes its purpose completely evident	44	49	89.8
No distracting information or content	31	40	77.5
**Word Choice and Style**			
Common, everyday language	48	49	98.0
Medical terms are defined and used only to familiarise readers	46	49	93.9
Active voice	47	49	95.9
**Use of Numbers**			
Numbers are clear and easy to understand	20	23	87.0
Does not expect readers to do calculation	39	40	97.5
**Organisation**			
Information is broken down into short sections	32	43	74.4
Sections have informative headers	43	43	100.0
Presents information in a logical sequence	49	49	100.0
Provides a summary	12	43	27.9
**Layout & Design**			
Uses visual cues on key points	41	42	97.6
Text on screen is easy to read	7	7	100.0
Allows user to hear the words clearly	5	5	100.0
**Use of Visual Aids**			
Uses visual aids whenever possible	13	40	32.5
Visual aids reinforce rather than distract	12	13	92.3
Visual aids have clear titles and captions	12	12	100.0
Visual aids are clear and uncluttered	33	34	97.1
Tables are simple with short, clear role and column headings	9	9	100.0
**Actionability**^b^	**26**	**49**	**53.1**
Identifies at least one action for the user	49	49	100.0
Addresses the user directly	49	49	100.0
Breaks down actions into explicit steps	45	49	91.8
Provides tangible tools whenever it could help	15	40	37.5
Instructions and examples for calculations	0	2	0.0
Explains how to use the charts, diagrams etc	8	10	80.0
Use visual aids whenever possible to help users act on instructions	10	40	25.0

^a^Some items were not applicable to all texts (e.g. an item may apply only to audio-visual materials). Percentages represent the proportion of relevant texts for which the item was endorsed

^b^Counts for overall understandability and actionability reflect the number of resources for which 70% of corresponding applicable items were endorsed

Of the 49 resources, about half had adequate actionability (*n* = 26, 53.1%). Although all resources could identify at least one action for the user and addressed the reader directly, very few provided tangible tools (*n* = 15, 37.5%) or visual aids to help users act on instructions (*n* = 10, 25.0%).

Based on median SHeLL Health Literacy Editor assessment scores, a typical text was written at about a grade 12 reading level and used the passive voice 6 times. About one in five words in a typical text were considered complex (19%) (Table 3).

**Table 3:** SHeLL Heath Literacy Editor assessments, *n* = 49

Assessment	Median	IQR	n (%) within target^a^
Grade reading score	12	3	4 (8)
Complex language (%)	19	9.7	14 (29)
Instances of passive voice	6	13	12 (25)

^a^Grade 8 or lower, < 15% complex language, < 2 instances of passive voice

### Stage 3: Workshops to review and interpret audit results, and identify priority areas

In Workshop B, study authors presented the results of the health literacy audit (Stage 2). Twenty-five attendees reflected on the results as a whole and discussed in further detail in small groups. This workshop comprised 12 consumers including the 5 consumers from Workshop A; 12 staff; and 1 health literacy researcher. Four of the attendees were also study authors (1 consumer, 2 staff, 1 health literacy researcher). Demographic characteristics of consumers are shown in Table 4. In addition, consumers represented either direct lived experience or a close personal connection to culturally and linguistically diverse communities, Aboriginal and Torres Strait Islander communities, younger people, carer roles, LGBTQI + , disability, homelessness, and people living with chronic conditions. Staff who attended the latter workshops (B and C) included those at executive and management levels.

**Table 4:** Demographics of consumers who took part in workshops

Characteristics	N (%)
**Gender**	
Male	2 (17)
Female	10 (83)
**Age group (years)**	
< 40	1 (8)
40–50	1 (8)
50–60	3 (25)
> 60	6 (50)
**Regionality**	
Metropolitan	8 (75)
Regional	3 (25)
**Language spoken at home**	
English only	11 (92)
Another language	1 (8)
**Aboriginal or Torres Strait Islander**	
Yes (Aboriginal)	1 (8)
No	11 (92)
**Education**	
University	7 (58)
Less than university level	5 (42)
**Total**	**12**

Discussions centred on how to interpret the audit results to identify priorities for revising or adapting existing content. This included time that was allocated to identify potential ‘gaps’ that new content could address (for example, for more specific target audiences, and content areas or health literacy skills that could be more prominent). In Workshop C, attendees formed four small groups. Each group was given two resources assessed as having poor actionability, grade reading score, complex language, and passive voice. Each group was asked to reflect on how their specific resources could be further improved for use by consumers.

**Fig. 2:**
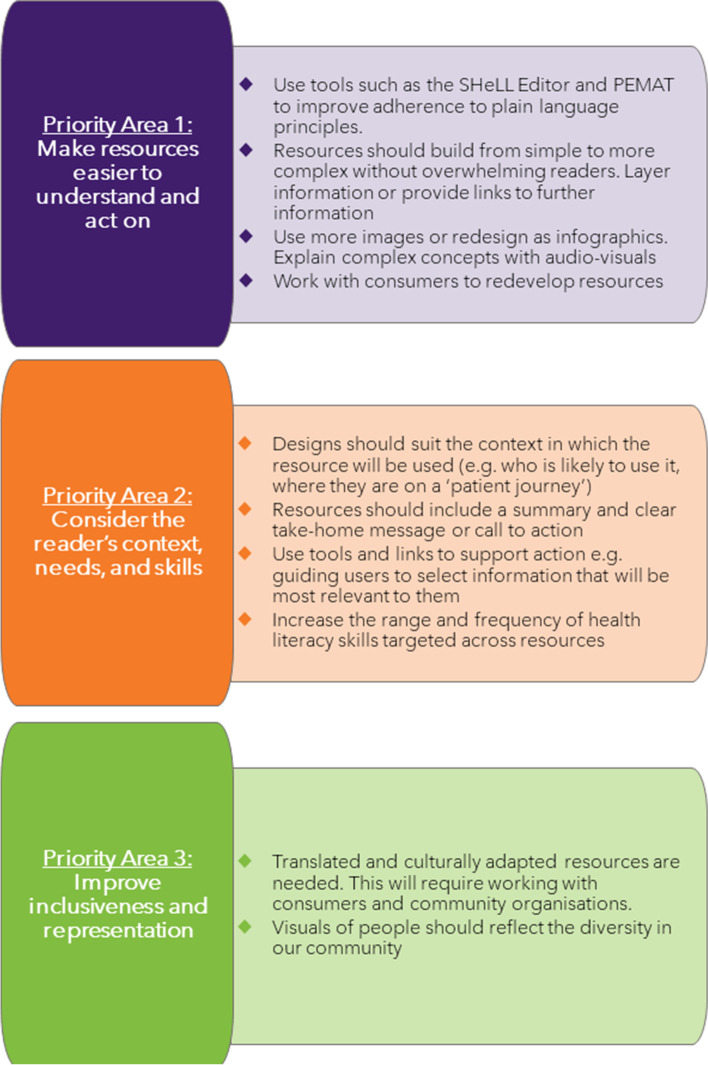
Key priority areas identified from health literacy audit activities

Figure 2 depicts the three key priority areas identified at the end of workshop C. The first two priority areas were more closely related to the PEMAT and SHeLL Editor results, whilst the third relates more closely to discussions about potential gaps in the resources. Workshop discussions helped shape these priority areas. For example, health literacy assessments indicated that the health information was often too complex (see Stage 2). Consumers discussed the importance of offering simple information alongside more detailed information. They suggested that layering information could achieve this goal, as well as using audio-visual formats for more complex concepts.

Similarly, the PEMAT assessments from Stage 2 identified that many resources had poor actionability because they lacked tangible tools or visual aids. Consumers emphasised that tangible tools and visual aids would have limited utility if the purpose of a resource was unclear to readers, including the context in which it should be used.

### Stage 4: Critical reflections and feedback on the audit process

Three staff and eight consumers took part in the interviews, including the four consumers involved in the PEMAT assessments. Participants appreciated the opportunity to be involved in the audit and highlighted four key ways to further improve the audit process (Table 5).

**Table 5:** Qualitative feedback for improving the audit process

Opportunity for improvement of audit process	Description	Supporting quotes
**1. Address issues relating to diversity of consumer representation**	• The diversity of workshop members’ experiences (see description in Stage 3) was not formally described to workshop attendees	“When you think about it … we're pretty savvy consumers… I did wonder about, how our [PEMAT] responses might actually stand up in the real world… what actually is meaningful for [people from other communities or with other health conditions] may be completely different for me…” (consumer involved in PEMAT assessments and workshops)
	• Many of the consumers perceived a need for a more diverse range of consumers in the project. For experienced consumers it could sometimes be challenging to speak on behalf of other consumers	
**2. Provide greater opportunity for unstructured feedback and set clear expectations about the consumers’ role in the project**	• Some participants wanted to give more detailed input on individual resources that went beyond the scope of the assessment tools and time constraints of the workshop discussions	“I found the [PEMAT] tool restrictive in some ways because it couldn't actually grade the document on its on its real value because you are sort of constrained by the requirements of the tool… How is this document going to affect me? What am I going to learn from this that I don’t already know?” (consumer involved in PEMAT assessments and workshops)
	• This may reflect that perceptions that project tasks were unexpected. Consumers discussed that when they helped develop health information, they typically went through resources one-by-one	“I sit on [another] health literacy committee and… we just go through like 5 documents… we go through from top to bottom…not rushing through just because we want to check a box. That we did it, but it's actually holistic.” (consumer involved in workshops only)
	• In preparation for the workshops, all workshop attendees were provided with background material explaining the project’s scope and aims. However, consumer feedback suggested that this material was not well understood, or may not have been accessed	“…it might have been better to actually have had a lot more time and a bit more background on how what this actually was going to be presented… and to be more informed on the day when the group session actually happened.” (consumer involved in workshops only)
**3. Adapt the health literacy assessment tool to suit consumer needs**	• Consumers who used the PEMAT (Stage 2) saw value in having a systematic tool to assess health information	“I can understand that we do need to have it in some systematic way, but it can't be totally an academic system” (consumers involved in PEMAT assessments and workshops)
	• Resolving discussions with another consumer was considered a very useful and valuable part of the PEMAT assessment	“…the buddy system was really useful.” (consumer involved in PEMAT assessments and workshops)
	• They also discussed that the PEMAT could be more consumer-friendly, and quicker to use. This could include reducing the number of PEMAT items or adjusting item phrasing	
**4. Simplify presentations that summarise audit data and methods**	• Consumers and staff attending the first workshop felt that the PEMAT and SHeLL Editor results gave an interesting perspective	“I was quite surprised to see just how many of the resources didn't meet the criteria and had too high reading grades.” (Consumer involved in workshops only)
	• Consumers and some staff felt too much information was presented during the first workshop. This reduced allocated time for discussion	“I think the presentations were very clear and … I took away that people had a good handle on what was being done and what they needed to do as part of the workshops.” (Staff involved in workshops)
	• Some attendees at the first workshop felt that this information overload may have made it harder to achieve the aims and take ownership of the project	“I think the first workshop … was very long and quite intense… A lot of listening [about]… context that needed to be shared, but that just made that workshop an awful long time.” (Staff involved in workshops)
	• Participants were more positive about the second workshop and preferred the opportunity to discuss individual resources. This activity aligned closely with the expectations described in theme 2	

## Discussion

This paper presents a novel method for conducting large-scale consumer-centred health literacy audits. Consumers were involved throughout the process, from project planning and identifying which resources would undergo health literacy assessment, to conducting the health literacy assessments, interpreting results and identifying next steps. Three key areas for future action were identified: make resources easier to understand and act on; consider the readers’ context, needs, and skills; and improve inclusiveness and representation. Qualitative interviews highlighted that the audit method could be further improved by addressing issues related to diverse representation, providing greater opportunity for unstructured feedback, using a simpler subjective health literacy assessment tool, setting clear expectations about the project rationale and anticipated outcomes, and simplifying how audit data were presented.

Findings from this study add to the published literature about how to conduct a health literacy audit for a large existing database of health information resources. Previously, Alpert, Desens [17] conducted an audit that prioritised assessment of high-traffic health information resources (i.e. high page visits) within a US patient portal. The authors used data from a validated health literacy assessment tool to identify key overarching strategies to improve the quality of the patient portal’s health information. Building on this approach, the current study involved consumers throughout the process. These methods recognise the importance of understanding how health literacy needs and strengths relate to an organisation’s specific context, services, and actions [12], and the importance of partnering with consumers to deliver patient-centred health initiatives that have meaningful impact to the community [13, 14, 26].

Interviews also highlighted the need for a more consumer-friendly health literacy assessment tool. Although consumers perceived some value in the PEMAT’s systematic and comprehensive approach, ultimately they felt the tool was too lengthy, ‘academic,’ and inadvertently restricted the type of feedback they could provide. In theory, the PEMAT was designed for use by ‘lay’ people [20] as well as health literacy experts, and many of the items assess aspects of the text that are best suited to consumer feedback (e.g. ‘the material uses common, everyday language’). However, in practice, PEMAT assessments are rarely conducted by consumers. Further, to our knowledge this is the first study to report on the tool’s acceptability to consumers. Other existing health literacy assessment tools such as the CDC’s Clear Communication Index are likely to face similar issues, as they were not purpose-designed for consumers. Further work is needed to design and validate a quantitative health literacy assessment tool that applies a systematic and comprehensive approach to health literacy assessment, but is easier to use and more acceptable to consumers.

This study has several strengths in addition to strong consumer engagement. The health literacy audit incorporated a combination of subjective and objective health literacy assessments, including objective assessments that extend beyond grade reading score. This provided richer, more detailed quantitative data about the resources. Ultimately our findings demonstrate that consumer input is essential but alone may not be sufficient for ensuring that health literacy needs are met, as many of the existing resources did not adhere to health literacy guidelines even though consumers had been involved in their development. Second, audit data reported on the extent that resources supported health literacy skills relevant to quality use of medicines. This invited greater discussion about the organisation’s role in community capacity-building, an aspect of organisational health literacy that is often overlooked [2].

One of the key limitations was perceived lack of diversity amongst consumers. In Australia, there are several priority groups that do not receive or cannot easily access health information or health care [27, 28]. Meaningful partnerships with people from these communities is not only ethical; it is essential for developing and implementing equitable health literacy initiatives [12]. Lack of diversity in health consumers is a common issue, particularly with regards to culturally and linguistically diverse communities [14]. In this study, workshops attendees were of varied ages, location, and education; and many had direct or close personal connections to various priority groups. However, consumers discussed the need for greater diversity amongst workshop attendees. As such, the outputs of the workshops may have limited applicability to the various priority groups. Additional workshops with specific priority groups could help identify each group’s unique health literacy needs and strengths.

Another limitation was that workshops were conducted online because of the COVID-19 pandemic. Although this format has some advantages (e.g. reducing barriers related to travel or disability), it may have also contributed to perceptions that Workshop B was overwhelming and reduced opportunities to connect and build rapport. Lastly, there was a 6-month delay between the workshops and interviews. This may have resulted in low participation rates and the difficulty some participants had remembering details of the audit.

Since project completion, the organisation has taken several steps to act on findings from this audit and continue their strong consumer-centred approach. For example, consumers have led dissemination of findings at a research conference and continue to be involved in reviewing and updating the audited resources. The SHeLL Editor and PEMAT tool were embedded into standard document development and review processes within the organisation, with consumers contributing to staff training in the use of the PEMAT. Lastly, NPS MedicineWise strengthened partnerships with several peak bodies representing minority groups in efforts to increase representation from diverse groups. These are each practical examples of organisational health literacy actions that can inform the upcoming Australian National Health Literacy Strategy. In this study we focused on NPS MedicineWise’s direct-to-consumer health information. Health literacy audits of other content may benefit from engaging with additional relevant stakeholders, for example, health professionals, and relevant non-government and government organisations.

## Conclusion

This study reports novel methods for a consumer-centred large-scale health literacy audit. Findings highlight the clear value of involving consumers in assessing resources and interpreting audit data. For future iterations we recommend developing a consumer-centred health literacy assessment tool, increasing the diversity of consumer voices, and setting clear goals and expectations for each stage of the audit.

## Supplementary Information

**Additional file 1:** **Supplementary Table 1.** Descriptive characteristics of all resources, *N*=147. **Supplementary Table 2.** Descriptive health literacy characteristics of sample resources, *n*=49.

## Data Availability

All data generated or analysed during this study are included in this published article (and its supplementary information files).

## Abbreviations

CMI: Consumer Medicine Information
PEMAT: Patient Education Materials Assessment Tool
SHeLL: Sydney Health Literacy Lab
